# Ageism in Hiring Decisions: Sense of Purpose as a Mitigating Strategy

**DOI:** 10.1093/geroni/igab046.3561

**Published:** 2021-12-17

**Authors:** Megan Wilson, Patrick Hill

**Affiliations:** 1 Washington University in St. Louis, Saint Louis, Missouri, United States; 2 Washington University in St. Louis, St. Louis, Missouri, United States

## Abstract

Discrimination against older adults in the workplace is a pervasive issue that has important consequences for older adults, leading to lower well-being (Stokes & Moorman, 2020) and worse job outcomes (Macdonald & Levy, 2016). One area where discrimination manifests is in hiring practices, and thus research is needed to understand factors that impact willingness to hire older adults. One potential intervention target to reduce age discrimination in hiring is sense of purpose. Sense of purpose manipulations have previously been successful in increasing one’s comfort with diversity (Burrow & Hill, 2013), and thus may prove successful in combatting age discrimination in the workplace. Therefore, the current studies sought to understand whether sense of purpose was related to ageist attitudes and hiring decisions, and how a purpose manipulation might serve as a tool to combat discrimination in hiring. Across two studies (n = 594, MAge = 27.20), participants were shown the resumé of either an older adult applicant (62-years-old) or younger adult applicant (32-years-old), and were asked how hireable they would rate the applicant. The research found that the purpose manipulation did effectively increase individuals’ sense of purpose. In addition, the research found that sense of purpose was negatively related to ageist attitudes. However, the purpose manipulation was unsuccessful in reducing ageist attitudes, and had no effect on ageist discrimination in hiring. These results suggest that while sense of purpose is negatively related to ageist attitudes, manipulating purpose may not be an effective tool to reduce ageist attitudes or discrimination.

